# Cognitive function, health-related quality of life, and symptoms of depression and anxiety sensitivity are impaired in patients with the postural orthostatic tachycardia syndrome (POTS)

**DOI:** 10.3389/fphys.2014.00230

**Published:** 2014-06-25

**Authors:** Jake W. Anderson, Elisabeth A. Lambert, Carolina I. Sari, Tye Dawood, Murray D. Esler, Gautam Vaddadi, Gavin W. Lambert

**Affiliations:** ^1^Baker IDI Heart & Diabetes InstituteMelbourne, VIC, Australia; ^2^School of Psychology and Psychiatry, Monash UniversityMelbourne, VIC, Australia; ^3^Department of Physiology, Nursing and Health Sciences, Monash UniversityMelbourne, VIC, Australia; ^4^Central Clinical School, Faculty of Medicine, Nursing and Health Sciences, Monash UniversityMelbourne, VIC, Australia

**Keywords:** orthostatic intolerance, tachycardia, psychiatric comorbidity, sympathetic nervous system, noradrenaline transporter

## Abstract

The Postural Orthostatic Tachycardia Syndrome (POTS) is a condition in which heart rate increases abnormally when the individual assumes an upright position. In addition to the marked tachycardia, presyncope, and syncope, patients with POTS often complain of light-headedness, fatigue, and difficulty in concentrating. The present study assessed individuals with POTS for psychiatric comorbidity, anxiety sensitivity and health related quality of life and examined general cognitive ability. Data was obtained from patients with POTS (*n* = 15, 12 female, aged 30 ± 3 years) and age matched healthy subjects (*n* = 30, 21 female, aged 32 ± 2 years). Patients with POTS commonly presented with symptoms of depression, elevated anxiety and increased anxiety sensitivity, particularly with regards to cardiac symptoms, and had a poorer health related quality of life in both the physical and mental health domains. While patients with POTS performed worse in tests of current intellectual functioning (verbal and non-verbal IQ) and in measures of focused attention (digits forward) and short term memory (digits back), test results were influenced largely by years of education and the underlying level of depression and anxiety. Acute changes in cognitive performance in response to head up tilt were evident in the POTS patients. From results obtained, it was concluded that participants with POTS have an increased prevalence of depression and higher levels of anxiety. These underlying symptoms impact on cognition in patients with POTS, particularly in the cognitive domains of attention and short-term memory. Our results indicate that psychological interventions may aid in recovery and facilitate uptake and adherence of other treatment modalities in patients with POTS.

## Introduction

It is estimated that the lifetime cumulative incidence of syncope is approximately 35% (Ganzeboom et al., 2006) and that syncope-related hospitalizations cost the US healthcare system upwards of $2.4 billion (Sun et al., 2005). While most forms of orthostatic intolerance are associated with a reduction in blood pressure upon standing, one specific condition, the postural orthostatic tachycardia syndrome (POTS), is characterized by an excessive rise in heart rate (standing heart rate increases by 30 beats or more per min or exceeding 120 beats/min) and may be accompanied by presyncope or syncope in the absence of postural hypotension (Low et al., 1995; Jacob et al., 2000). The age of POTS patients is typically in the range 15–50 years, with women more likely to develop the disorder than men (Low et al., 1995). With research advances and growing physician education the number of patients found to have POTS has been rising.

In addition to the obvious cardiovascular-related symptoms, patients with POTS may describe difficulty in concentration and experiencing distraction and transient memory deficits which intrudes on personal and work life. Orthostatic stress in patients with chronic fatigue syndrome with POTS has been shown to be associated with neurocognitive impairment (Ocon et al., 2012). Repeated episodes of syncope may lead to distress and psychosocial dysfunction, seriously interfering with quality of life, and potentially increasing the incidence of mood or anxiety disorders.

The aim of the present study was to evaluate baseline psychological and cognitive functioning in patients with POTS and to examine whether individuals with POTS would exhibit an increased latency of response time on cognitive tasks when changing from the supine to head up position when compared with control participants.

## Materials and methods

### Participants

Patients with POTS (*n* = 15, 12 female, aged 30 ± 3 years) were recruited from either the medical outpatient clinics of the Alfred Hospital or the cardiovascular clinics at the Baker IDI Heart & Diabetes Institute. Diagnosis of POTS was made using criteria proposed by Low et al. (1995). Healthy subjects (*n* = 30, 21 female, aged 32 ± 2 years) were recruited from the general community. All participants underwent a comprehensive clinical and physical examination to screen for any previously undiagnosed medical conditions prior to their acceptance in the experimental protocols. Exclusion criteria included a history of major illness, current drug medication and current or previous use of psychotropic medication. The study was approved by The Alfred Hospital and Monash University Human Research Ethics Committees and all participants provided written, informed consent for their participation.

### Psychological evaluation and cognitive testing

All participants undertook a structured clinical interview in order to examine psychiatric co morbidity. All interviews were conducted by a trained psychologist. Evaluation included: Mini International Neuropsychiatric Interview (MINI, Version 5.0.0) (Sheehan et al., 1998); Hamilton depression and anxiety rating scales (HAM-D and HAM-A respectively) (Hamilton, 1960). The Anxiety Sensitivity Profile (ASP) was used to assess the patients' belief that their bodily sensations have harmful consequences (Taylor and Cox, 1998). The ASP is a 60-item expanded assessment of anxiety sensitivity and was designed to evaluate six relevant domains, although factor analytic studies suggest four first-order factors tapping fears of (1) respiratory, (2) cognitive dyscontrol, (3) gastrointestinal, and (4) cardiac symptoms. Each question relates to the likelihood that particular sensations would lead to something bad happening to the participant. Responses were provided as a likert score ranging from 0 (not likely at all) to 7 (extremely likely).

A selection of cognitive measures was chosen to measure intelligence and specific cognitive processes such as attention, information processing and short term memory. The major measure for general intelligence was the Wechsler Abbreviated Scale of Intelligence (WASI) (Wechsler, 1999). In addition, scales from the more comprehensive Wechsler Adult Intelligence Scale III (WAIS-III) were employed (Weschler, 1997). For example, the digit span subtest was included to examine attention and short-term memory. The digit span test is composed of two tasks administered independently of each other, namely digits forwards and digits backward. On both tasks the examiner read a series of number sequences to the examinee. For each digits forward item, the examinee was required to repeat the number sequence in the same order as presented. For digits backward the examinee was required to repeat the number sequence in the reverse order. Conceptually, the digit span subtest involves the auditory perception of simple stimuli, working memory to integrate and store information followed by simple vocal output of digits retained. Scores on digit span were referenced against normative data based on the WAIS-III where a scaled score of 10 ± 2 standard deviations is considered within the bounds of normal.

### Cognitive change in response to head-up tilt

The CogState computer based test battery (Maruff et al., 2009), comprising detection (attention), identification (information processing), and learning (short term memory) was used to assess dynamic changes in cognitive performance in response to orthostatic challenge. The detection task was used as a brief measure of psychomotor function, information processing or speed of thinking. For this task the participant was presented with a playing card face down at the center of the computer display. An on-screen wizard helper prompted the participant to “Press YES as soon as a card turns face up.” Thus, the participant would commence by pressing the “Yes” key. After a delay, the card in the center would then flip over so as to present face up. When this occurred the participant would be required to press the “Yes” key. This sequence was repeated multiple times, at randomly different intervals. The primary outcome measure of the detection task was speed of performance and the duration of the test was approximately 2 min. The identification task was employed as a measure of attention, choice reaction time, and decision making. A playing card was presented face down at the center of the computer display. The on-screen wizard prompted the participant: “Is the face-up card red?” The participant would respond by pressing the “Yes” key. Following a delay, the card in the center would then flip over so as to present face up. As soon as this occurred, the participant would be required to decide whether or not the card presented was red in color and subsequently indicate their choice by use of the “Yes” or “No” key. The primary outcome measure of the Identification task was speed and accuracy of this performance. Time taken to complete the task at each level of tilt was approximately 2 min. The One Card Learn test was used as a measure of short-term memory. A playing card was presented to participants face down at the center of the computer display. The on-screen wizard prompted the participant: “Have you seen this card before in this task?” They would then respond by pressing the “Yes” key. The card in the center would flip over so as to present face up. As soon as this occurred, the participant would then be required to decide whether or not the same card had appeared face up before in the task, and respond using the “Yes” or the “No” key (the first answer given in a test was always “No”). The card would then go to the back of the pack and after a random delay the next card would be revealed. As soon as the next card was revealed, the participants would indicate whether or not the card had been seen before in the task. The participant was required to attempt to recall all the cards that had been shown previously in the task in order to make a decision. Only some of the cards would repeat. Time taken to complete this task at each level of tilt was approximately 5 min.

Participants were familiarized with the testing program after which testing was performed with participants in the semi recumbent position and after 8 min of 60° head up tilt. Blood pressure was monitored continuously using the Finometer system (Finapress Medical System BV, Amsterdam, The Netherlands) and heart rate was extracted from lead-III ECG. All of these parameters were digitized with a sampling frequency of 1000Hz (PowerLab recording system, model ML 785/8SP, ADI Instruments). The double product, as an estimate of cardiac work, was calculated as the product of heart rate and systolic blood pressure.

### Quality of life assessment

The 36 Item Short-Form Health Survey (SF-36) was used to assess health related quality of life (Ware and Sherbourne, 1992). The questionnaire contains 36 items that yield 8 category scales: physical functioning, role limitations caused by physical problems, bodily pain, general health, vitality, social functioning, role limitations caused by emotional problems, and mental health. Scale scores range from 0 to 100, with higher scores indicating better health. The 8 category scores can be aggregated into two summary scales, the physical component summary scale and the mental component summary scale. The SF-36 has demonstrated excellent psychometric properties in both patient and healthy control populations (Ware and Sherbourne, 1992).

### Statistical analysis

Statistical analysis was performed using Statistical Package for the Social Sciences (SPSS) Version 22.0 (SPSS Inc., Chicago USA). Group data are reported as mean ± standard error of the mean. Comparisons between groups were performed using a One Way ANOVA. Analysis was performed on ranks for non-Gaussian data. The effects of head up tilt were assessed using One Way ANOVA for repeated measures. Associations between the variables were examined using Pearson product moment correlation and stepwise multiple regression. A value of *P* < 0.05 was considered significant.

## Results

### Participant demographics, depression and anxiety scores

The baseline clinical characteristics, years of education and depression and anxiety scores of the patients with POTS and the control subjects are presented in Table 1. The control group was well matched for age, gender, and BMI. Patients with POTS tended to have spent approximately 1½ less years in formal education. Supine heart rate, blood pressure and, hence, double product, were similar between groups. Clinician rated Hamilton anxiety and depression ratings were significantly higher in the POTS group. Thirteen of the subjects with POTS presented with current mild-moderate major depressive disorder and one had a past history of panic disorder. In the control group one subject presented with symptoms of depression, with the severity being in the mild range (Ham-D 8-13), and one subject had symptoms of generalized anxiety disorder. There was no association between years of education, Ham-D or Ham-A scores and resting heart rate or blood pressure.

**Table 1:** **Participant characteristics**.

	**Control**	**POTS**	***P* value**
Age (years)	31.6±1.8	30.1±2.9	0.66
BMI (kg/m^2^)	25.6±0.7	24.6±1.1	0.45
Heart rate (b/min)	71±3	74±3	0.37
Blood pressure (mmHg)	130±3/75 ±2	132±5/75 ±3	0.76/0.94
Double product (mmHg.b/min)	9292±456	9849±669	0.52
Education (years)	14.6±0.6	12.9±0.6	0.05
Ham-A score	5±1	18±2	<0.001
Ham-D score	3±1	12±1	<0.001

### Health related quality of life

**Figure 1:**
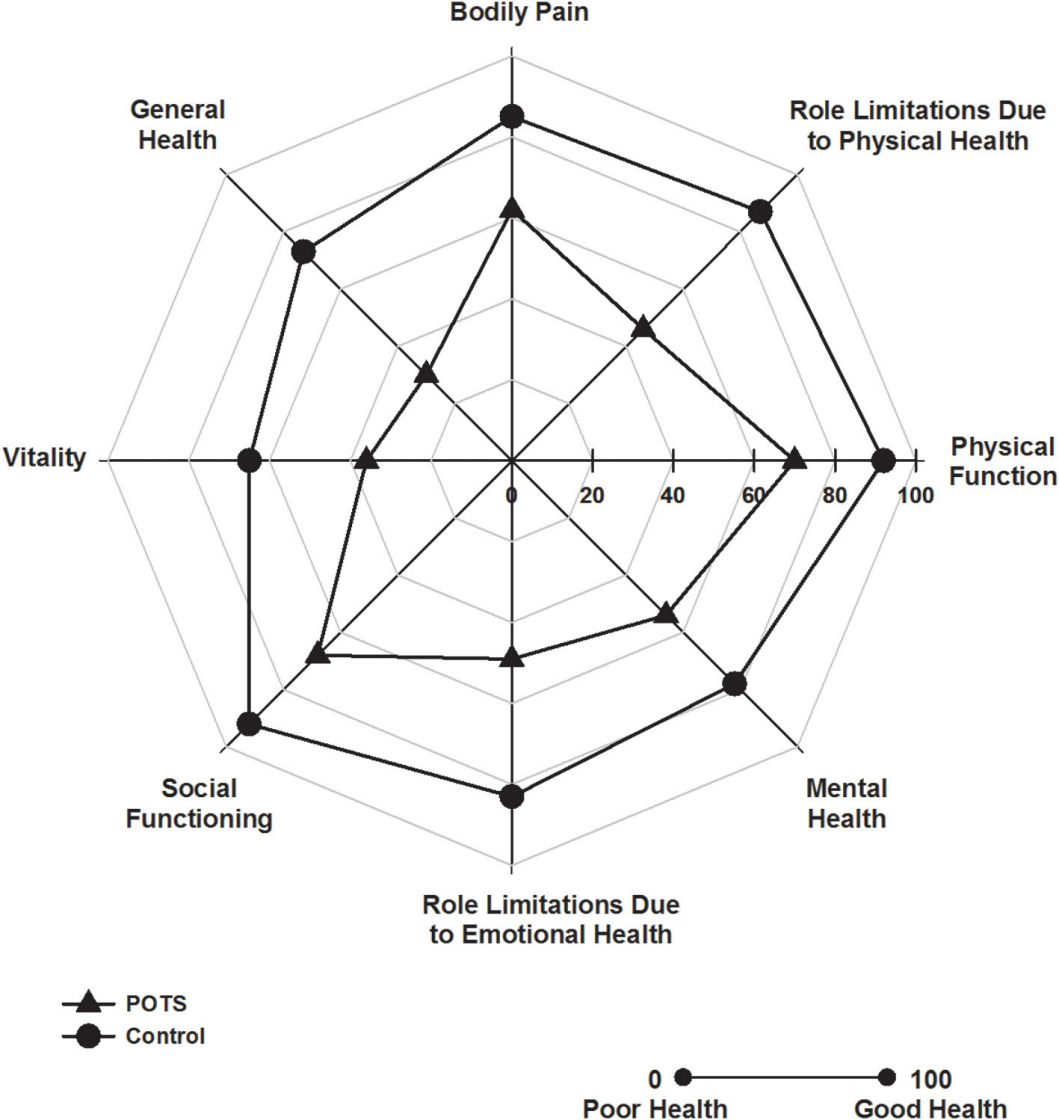
**Health related quality of life**. Polar plot indicating SF-36 domain scores in patients with POTS (▲) and in healthy subjects (•). Patients with POTS scored significantly worse in all domains, *P* < 0.001, *n* = 30 control and 15 POTS.

Patients with POTS scored significantly worse in all of the eight SF-36 domain scores (Figure 1, all *P* < 0.001). Consequently, both the physical (52 ± 6 vs. 41 ± 7, *P* < 0.001) and mental health (51 ± 8 vs. 39 ± 14, *P* < 0.001) component summary scores were significantly diminished in the subjects with POTS. There was no association between resting heart rate or blood pressure and any of the SF-36 measures.

### Anxiety sensitivity

Anxiety sensitivity is presented in Table 2. Participants with POTS reported increased anxiety sensitivity in relation to cognitive and cardiac symptoms, and dissociation (Table 2). The ASP Full Scale score was significantly worse in the POTS patients. Anxiety sensitivity was not quantitatively linked to heart rate or blood pressure.

**Table 2:** **Anxiety sensitivity profile (ASP) scores across multiple domains of symptom anxiety sensitivity**.

	**Control**	**POTS**	***P* value**
Cognitive	19±3	37±4	0.04
Cardiac	16±3	25±3	<0.001
Gastro	14±2	17±3	0.32
Respiratory	22±3	33±5	0.06
Humiliation	17±3	23±3	0.16
Dissociation	18±3	27±2	0.03
ASP full (/420)	107±15	158±17	0.02

### Cognitive function

Neuropsychological assessment data in healthy subjects and POTS participants are presented in Table 3. Univariate analysis indicated that patients with POTS performed significantly worse in all aspects examined. While performance in the cognitive tests was not influenced by age or BMI there occurred strong associations between test results and years of education (positive, Table 3, all *P* < 0.01) and underlying level of depression and anxiety (both negative, Table 3, all *P* < 0.01). Forward stepwise regression analysis indicated that the verbal IQ test results could be predicted by a combination of years of education (*P* = 0.02) and Ham-A rating (*P* = 0.04), with the combination of these factors accounting for approximately 30% of the variance in the verbal IQ test results (Table 4). Non-verbal (performance) IQ could be predicted from years of education (*P* < 0.001). Full scale IQ could be predicted by a combination of years of education (*P* = 0.004) and Ham-A rating (*P* = 0.01), with these factors accounting for 43% of the variance in the Full scale IQ test results (Table 4). Performance in the digits forward and backwards tests was influenced more by the underlying level of depression than by years of education or anxiety. Digit span forward results could be predicted by a combination of Ham-D score (*P* = 0.003) and years of education (*P* = 0.02), with the combination of these factors accounting for 41% of the variance in digits forward scores. Hamilton depression scores accounted for 27% of the variance in digits backwards scores (*P* < 0.001). The overall Digit span score could be predicted by a linear combination of Ham-D score (*P* < 0.001) and years of education (*P* = 0.01), with the combination of these factors accounting for 46% of the variance in the test score (Table 4).

**Table 3:** **Cognitive profile in healthy subjects and in patients with POTS**.

**Test**	**Control**	**POTS**	***P* value**	**Education (Pearson *r*)**	**Ham-D (Pearson *r*)**	**Ham-A (Pearson *r*)**
Verbal IQ (VIQ)	110±2	102±2	0.02	0.50	−0.46	−0.47
VIQ %	71±4	52±6	0.01	0.53	−0.52	−0.53
Non-verbal-IQ (NVIQ)	114±2	106±3	0.01	0.52	−0.42	−0.46
NV-IQ %	78±3	60±6	0.006	0.55	−0.44	−0.49
Full scale IQ (FSIQ)	114±2	33±5	0.003	0.58	−0.51	−0.56
FSIQ %	78±3	55±6	0.001	0.64	−0.58	−0.65
Digit span (forward)	11.0±0.4	8.9±0.4	0.001	0.55	−0.59	−0.56
Digit span (backward)	8.1±0.3	6.3±3	0.003	0.47	−0.53	−0.52
Digit span	19±1	15±1	0.001	0.57	−0.63	−0.60
Digits scaled score	11.2±0.4	8.9±0.6	0.001	0.51	−0.62	−0.62

Verbal IQ, verbal intelligence quotient; Non-verbal IQ, non-verbal intelligence quotient; Full Scale IQ, full scale intelligence quotient, Digit span presented as number of digits recalled, scaled scores of WASI where population X = 10; IQ scores where population X = 100; % = percentile score relative to normative data of WASI. Hamilton Depression rating (Ham-D), Hamilton Anxiety rating (Ham-A). Analyses controlled for age and gender.

**Table 4:** **Multivariate analysis of variables influencing cognitive test results**.

**Test**	**Variables**	**β**	***P***
**VERBAL IQ (VIQ)**			
Model 1	Education	0.50	<0.001
Model 2	Education	0.35	0.02
	Ham-A	−0.31	0.04
**NON-VERBAL-IQ (NVIQ)**			
Model 1	Education	0.52	<0.001
**FULL SCALE IQ (FSIQ)**			
Model 1	Education	0.58	<0.001
Model 2	Education	0.41	0.004
	Ham-A	−0.35	0.01
**DIGIT SPAN (FORWARD)**			
Model 1	Ham-D	−0.59	<0.001
Model 2	Ham-D	−0.42	0.003
	Education	−0.34	0.02
**DIGIT SPAN (BACKWARD)**			
Model 1	Ham-D	−0.54	<0.001
**DIGIT SPAN**			
Model 1	Ham-D	−0.63	<0.001
Model 2	Ham-D	−0.46	0.003
	Education	−0.34	0.01

Verbal IQ, verbal intelligence quotient; Non-verbal IQ, non-verbal intelligence quotient; Full Scale IQ, full scale intelligence quotient, Digit span presented as number of digits recalled. Other variables included in analysis: group (i.e., Control vs. POTS), age and gender.

### Cognitive function in response to head up tilt

**Figure 2:**
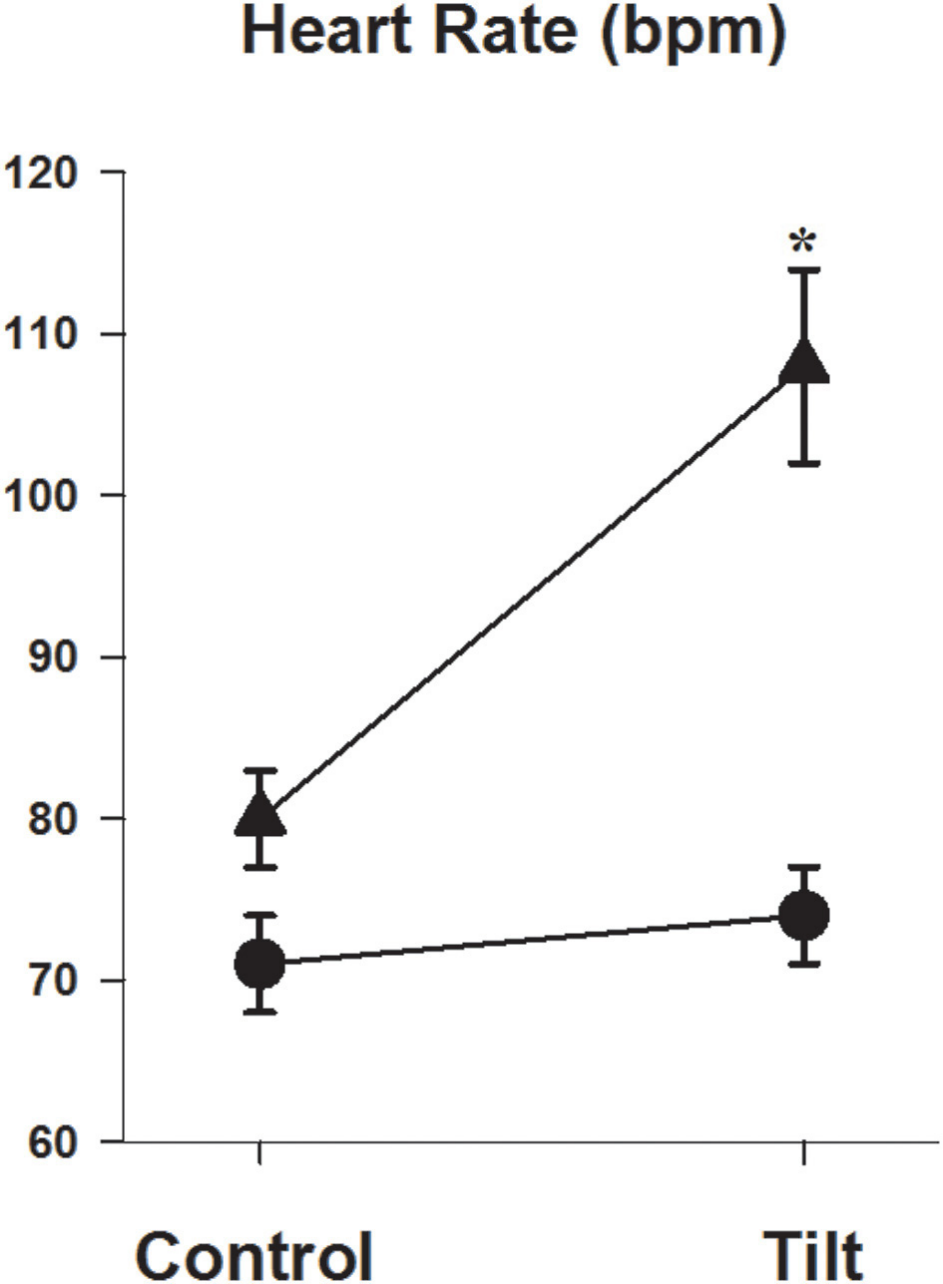
**Heart rate response during head up tilt**. Graph showing heart rate in the semi recumbent position and at 60° head up tilt in patients with POTS (▲) and in healthy subjects (•). ^*^P < 0.05, *n* = 26 control and 11 POTS.

The change in heart rate in response to 60° head up tilt was significantly greater in the POTS patients (11 ± 1 vs. 34 ± 3 b/min, *P* < 0.001, Figure 2). Similarly, the double product in POTS patients increased substantially following head up tilt (+890 ± 839 vs. +5139 ± 655 mmHg.b/min, *P* = 0.008). There was no difference in the blood pressure response during tilt testing (141 ± 4/88 ± 3 in control vs. 136 ± 5/83 ± 4 mmHg in POTS, *P* = 0.58/0.43).

**Figure 3:**
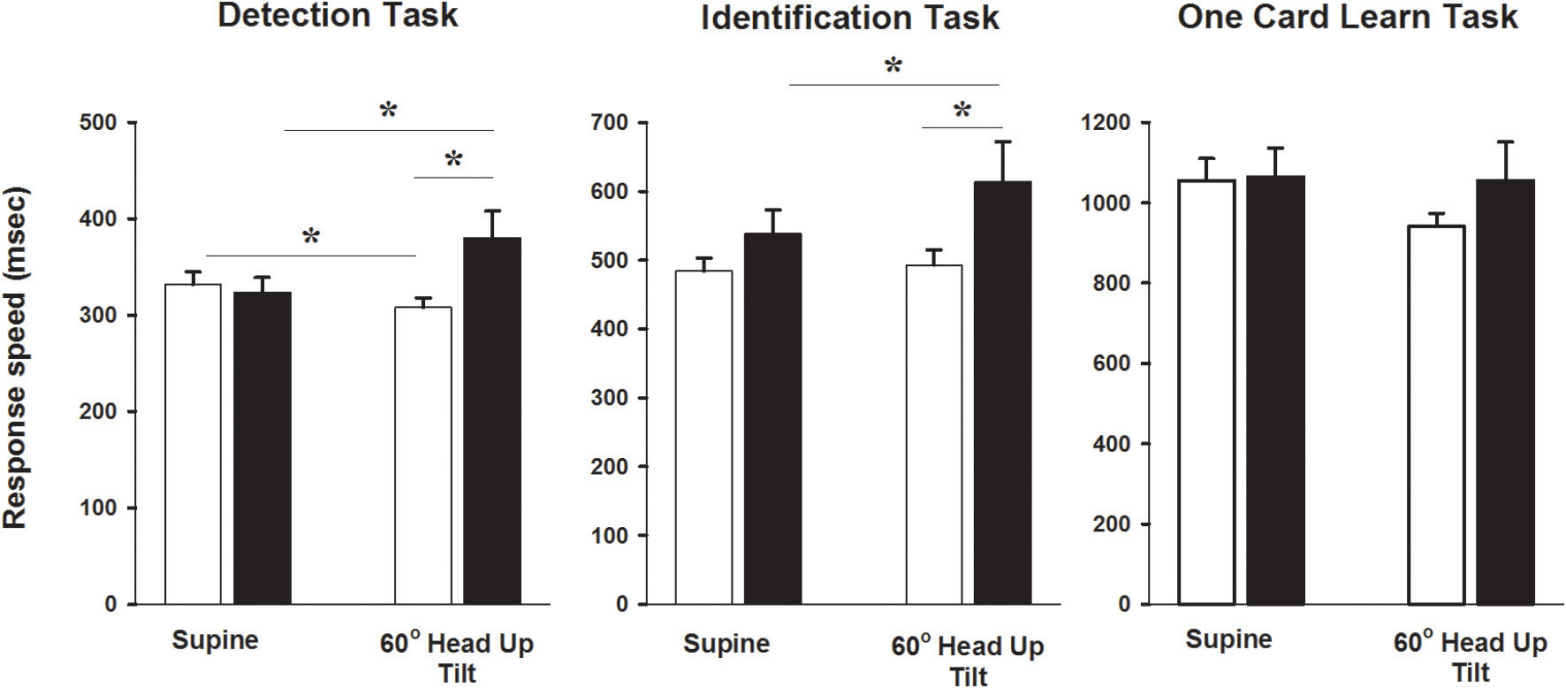
**Cognitive testing during head up tilt**. Bar graphs showing CogStat test results in the semi recumbent position and at 60° head up tilt in patients with POTS and in healthy subjects. ^*^P < 0.05, *n* = 26 control and 11 POTS. Black bars indicate patients with POTS and white bars indicate healthy subjects.

Acute changes in cognitive function, with recordings performed in the semi recumbent position and at 60° head up tilt, were examined in 26 of the control subjects and in 11 POTS patients. In the semi recumbent position there were no differences between groups in attention (detection task response speed 332 ± 13 vs. 325 ± 15 ms, Control vs. POTS, *P* = 0.76), information processing (identification task processing speed 484 ± 19 vs. 538 ± 35 ms, Control vs. POTS, *P* = 0.19) or short term memory (1055 ± 55 vs. 1064 ± 72 ms, Control vs. POTS, *P* = 0.94, Figure 3). Patients with POTS performed significantly worse than the control subjects in the attention (detection) test during 60° head up tilt (Figure 3). *Post-hoc* analysis indicated that there was a 15% improvement in performance time between supine and tilt 60° in healthy subjects (*P* < 0.05), and that there was a significant worsening of performance in individuals with POTS, as indicated by a 24% increase in latency of performance response times between the semi recumbent position and tilt 60° (*P* < 0.05). The change in detection task response time following tilt was significantly associated with the change in heart rate (*r* = −0.51, *P* = 0.004). In individuals with POTS there occurred a 13% increase in response time between the semi recumbent position and 60° head up tilt with the identification task, resulting in a 27% difference in performance response times between both groups at tilt 60°. The change in identification task response time could be predicted by a combination of full scale IQ (*P* < 0.001) and the SF36 social (*P* = 0.007) and physical (*P* = 0.03) functioning scores, with the combination of these factors accounting for 47% of the variance in the change in identification task response time. Performance in the one card learning task was not influenced by tilt.

## Discussion

In this study we examined cognitive function in patients with POTS, paying particular attention to the possible association between cognitive performance and underlying symptoms of depression and anxiety. Patients with POTS commonly presented with symptoms of depression, elevated anxiety and increased anxiety sensitivity, and had a poorer subjective health related quality of life in both the physical and mental health domains. While patients with POTS performed worse in tests of current intellectual functioning (verbal and non-verbal IQ) and in measures of focused attention (digits forward) and short term memory (digits back), test results were influenced largely by years of education and the underlying level of depression and anxiety rather than the categorical diagnosis of POTS. Acute changes in cognitive performance in response to head up tilt were evident in the POTS patients.

Patients with POTS experienced markedly diminished health-related quality of life across both physical and mental health domains. This observation is congruent with findings of a reduction in quality of life in the context of repeated episodes of syncope (Linzer et al., 1991) and in those with chronic illness (Lambert et al., 2012). It is recognized that the prevalence of depression is elevated in patients with chronic, physical illnesses such as heart disease, stroke, cancer, and diabetes mellitus (Clarke and Currie, 2009). In line with this observation we found that the majority of our POTS patients presented with symptoms of major depressive disorder and had an elevated level of anxiety. Raj and colleagues noted that, at the time of testing, patients with POTS presented with symptoms of mild depression, although life time prevalence of major depressive disorder was not elevated (Raj et al., 2009b), and McGrady et al. found that subjects who had a positive tilt table test for autonomic dysfunction had higher depression scores (McGrady et al., 2001). Importantly, in our study the increased level of depression and anxiety impacted negatively on some aspects of cognitive function, in particular attention and short-term memory. Depressive symptoms have been associated with distractibility and impaired cognitive processing, particularly in the context of higher cognitive abilities such as executive processing, organization, and recall of information. Our data is consistent with a previous report by Raj et al. who demonstrated that patients with POTS scored worse than controls on Connors Adult Attention Deficit Hyperactivity Disorder (ADHD) inattention/memory problems subscale (Raj et al., 2009b). It is likely that the association between depression and cognitive performance reflects the underlying neurobiology of depression, with alterations in brain monoamines influencing both depression and cognition (Lambert et al., 2000; Austin et al., 2001; Schmitt et al., 2006; Barton et al., 2008; Wingen et al., 2008; Chalermpalanupap et al., 2013). Noradrenaline transporter (NET) dysfunction is evident in patients with POTS (Shannon et al., 2000; Lambert et al., 2008) and in untreated patients with major depressive disorder (Barton et al., 2007). Interestingly, genetic variation in the NET gene has been associated with deficits in memory and attention in patients with ADHD (Thakur et al., 2012). Although coding mutations in the NET gene in POTS are rare (Shannon et al., 2000) epigenetic modification of the NET gene may impact NET expression in POTS (Bayles et al., 2012) and perhaps influence learning and memory (Day and Sweatt, 2011).

An abnormality in neuronal noradrenaline reuptake could sensitize the heart to sympathetic nervous activation. Interestingly, given that POTS is more prevalent in females, previous studies have noted differences in NET function between genders. For instance, examination of the acute effects of selective NET blockade with reboxetine indicated that NET function, or expression, is reduced in women (Schroeder et al., 2004). Moreover, female sex hormones influence the cardiovascular response to NET inhibition in young women (Moldovanova et al., 2008) and estrogen supplementation attenuates the sympathetic and hemodynamic response to mental stress in both elderly men and perimenopausal women (Komesaroff et al., 1999, 2002). An earlier report by Rosano et al. noted a reciprocal relationship between female sex hormones and the incidence of paroxysmal supraventricular tachycardias (SVT), with an inverse correlation between plasma 17-β-estradiol and a positive association between plasma progesterone and number of episodes and duration of SVT (Rosano et al., 1996). Although muscle sympathetic activity and the sensitivity of the sympathetic baroreflex vary throughout the menstrual cycle in healthy subjects (Minson et al., 2000), Fu et al. found that the plasma catecholamine response to standing remained unchanged during the phase of the menstrual cycle but noted an association between incidence of presyncope and reduced plasma renin activity and aldosterone levels in the early follicular phase of the menstrual cycle in patients with POTS (Fu et al., 2010). Interestingly, changes in cognitive function during the menstrual cycle have also been reported. Changes in estrogen levels have been shown to be associated with improved verbal working memory span (Rosenberg and Park, 2002), with effects perhaps being controlled by prefrontal dopaminergic neuronal activity (Jacobs and D'esposito, 2011). Although we found no effect of gender on cognitive function, our sample size was small and we did not take into account phase of the menstrual cycle or the use of oral contraceptives in our investigation. Whether cyclic variations in female sex hormones influenced the results of the present study remains unknown but merits further attention.

Given the distinctive feature of POTS, namely the marked elevation in heart rate on standing, increased anxiety sensitivity, particularly in relation to cardiac features is perhaps not surprising and is consistent with some (Benrud-Larson et al., 2002, 2003), but not all (Raj et al., 2009b), previously reported data. Individuals with disorders of postural syncope may be more likely to focus attention on the negative consequences of fainting; a preoccupation which subsequently contributes to reduction in perceived health related quality of life (McGrady et al., 2001). While physical symptoms are likely to be important factors in reducing quality of life in POTS, our results also indicate that heightened incidence and severity of psychological and cognitive factors may also play a role. Hyper vigilance to somatic sensations and catastrophic cognitions have been associated with greater limitations on day to day activities and increased perception of disability (Benrud-Larson et al., 2003).

We found that patients with POTS experienced delays in attention and information processing compared with the control subjects during head up tilt. This observation is consistent with a recent report by Stewart et al. (2012) who examined cognitive performance in young chronic fatigue syndrome patients with POTS. Perhaps underpinning the acute impairment in cognitive performance, reduced cerebral blood flow and a possible uncoupling of cerebral autoregulation during head up tilt has been demonstrated in patients with POTS (Ocon et al., 2009). Additionally, given that the reduction in cognitive performance during the detection task was significantly associated with the magnitude of the change in heart rate, and that patients with POTS displayed significantly elevated anxiety sensitivity in relation to cardiac symptoms, it is possible that, in the context of increased vigilance to somatic sensations, attention is diverted and impaired cognitive performance follows.

Treatment of POTS involves the implementation of a disease management plan encompassing education, pharmacotherapy, and exercise. Remaining hydrated, avoidance of excessive heat, wearing compression tights in order to reduce venous pooling, administration of fluid retaining agents, low dose propranolol, acetylcoholinesterase inhibitors and exercise are all important elements of the POTS treatment arsenal (Raj et al., 2005, 2009a; Fu et al., 2011; Shibata et al., 2012). Results from our study point also to a possible benefit of psychological interventions. Techniques facilitating stress reduction and cognitive behavior therapy, with an emphasis on identifying and restructuring unhelpful beliefs, primarily those catastrophising consequences of physical symptoms from standing, may aid in recovery and facilitate uptake and adherence of other treatment modalities.

### Conflict of interest statement

The laboratories of Professors Lambert and Esler currently receive research funding from Medtronic, Abbott Pharmaceuticals, Servier Australia and Allergan. Professor Lambert has acted as a consultant for Medtronic and has received honoraria or travel support for presentations from Pfizer, Wyeth Pharmaceuticals, Servier and Medtronic. Professor Esler serves on scientific advisory boards of Abbott (formerly Solvay) Pharmaceuticals and Medtronic. The authors declare that the research was conducted in the absence of any commercial or financial relationships that could be construed as a potential conflict of interest.
